# Prediction of ground reaction forces while walking in water

**DOI:** 10.1371/journal.pone.0219673

**Published:** 2019-07-18

**Authors:** Alessandro Haupenthal, Heiliane de Brito Fontana, Daniela Pacheco dos Santos Haupenthal, Marcel Hubert, Helio Roesler, Caroline Ruschel

**Affiliations:** 1 Department of Physiotherapy, Federal University of Santa Catarina, Araranguá, SC, Brazil; 2 School of Biological Science, Federal University of Santa Catarina, Florianópolis, SC, Brazil; 3 Health Science Unit University of Southern Santa Catarina, Criciúma, SC, Brazil; 4 Aquatic Biomechanics Research Laboratory, Health and Sports Science Centre, University of the State of Santa Catarina, Florianópolis, SC, Brazil; Nanyang Technological University, SINGAPORE

## Abstract

Despite being a key concept in rehabilitation, controlling weight-bearing load while walking, following lower limb injury is very hard to achieve. Walking in water provides an opportunity to prescribe load for people who have pain, weakness or weight bearing restrictions related to stages of healing. The aim of this experimental study was to evaluate and validate regression models for predicting ground reaction forces while walking in water. One hundred and thirty seven individuals (24±5 years, 1.71±0.08 m and 68.7±12.5 kg) were randomly assigned to a regression group (n = 113) and a validation group (n = 24). Trials were performed at a randomly assigned water depth (0.75 to 1.35 m), and at a self-selected speed. Independent variables were: immersion ratio, velocity, body mass, and waist, thigh and leg circumferences. Stepwise regression was used for the prediction of ground reaction forces and validation included agreement and consistency statistical analyses. Data from a force plate were compared with predicted data from the created model in the validation group. Body mass, immersion ratio, and velocity independently predicted 95% of the vertical and resultant ground reaction force variability, while, together, velocity and thigh circumference explained 81% of antero-posterior ground reaction force variability. When tested against the data measured in validation samples, the models output resulted in statistically similar values, intraclass correlation coefficients ranging from 0.88 to 0.90 and standard errors of measurement, 11.8 to 42.3 N. The models introduced in this study showed good predictive performance in our evaluation procedures and may be considered valid in the prediction of vertical, antero-posterior and resultant ground reaction forces while walking in water. All predictive variables can be easily determined in clinical practice. Future studies should focus on the validation of these models in specific populations.

## Introduction

After a lower limb injury or surgery, proprioception, strength, balance and range of motion are often impaired [1–3]. These changes, sometimes accompanied by pain, impair movement control [4,5], pose a challenge to the introduction of exercises that involve body weight load. Even after familiarization and training with gait assistive devices such as crutches, persons with a lower limb injury are not able to adhere to the maximum load prescribed in rehabilitation [6,7]. As an alternative, physiotherapists have opted for aquatic exercises [8–10]. In water, physiotherapists can choose training parameters that may help with the control of ground reaction forces, even in the presence of poor movement control.

Ground reaction forces (GRFs) are reduced while walking in water when compared to walking on dry land [11,12]. Specifically, the vertical component peaks are substantially reduced, and the antero-posterior component changes in pattern but not much in peak magnitude [11,12]. Currently it is unclear whether these changes occur homogenously in a large population and whether controlling external exercising parameters while walking in water is effective in determining GRFs while walking.

The greater density of water compared to air leads to a much greater drag force in water. The resistance, present throughout the entire movement, increases as the velocity increases and the frontal body projected area gets larger [11,13,14]. In water, therefore, there is a drag force that strongly depends on exercise parameters and individuals’ body shape [13].

In addition, the volume occupied by the body mass will influence buoyancy. Body density is lower than water density, resulting in our bodies floating. The magnitude of the buoyant force acting on the body during head-out exercises is equal to the weight of the water volume displaced by the body [15]. Therefore velocity, water depth and individual anthropometric data are important determinants of GRFs while walking in water. While the effects of velocity [11] and water depth [11,13] have been previously explored, our knowledge of the effect of individual anthropometric characteristics is limited. [13,16]

Despite the increase in the number of investigations of aquatic biomechanics [16–22] and the specialized instrumentation now used for underwater measurements [23], a gap still exists between the knowledge available from the literature and current practice when choosing exercise parameters during water rehabilitation. In order to i) allow a better understanding of the relationship between ground reaction forces while walking in water and the related modifying factors, and ii) provide a potentially useful tool to control these forces in clinical practice, we aimed to develop and validate statistical regression models for the prediction of GRFs while walking in water in a healthy population.

We hypothesized that a statistical model based on water depth, walking velocity and subject anthropometrical characteristics is a valid and precise tool in the prediction of GRFs while walking in water. The quantification of GRF response to these factors may provide a better rationale for the prescription of walking in water to a person with a lower limb injury who requires weight bearing control [24–28].

## Materials and methods

This was an experimental study. Subjects were based on the following criteria: (a) aged between 18 and 40 years (b) absence of pain, injuries, or surgeries in the last six months, (c) confident in water (d) absence of contraindications to being immersed [29]. The minimum sample size was estimated as 105 participants based on 7 variables and 15 data points per variable [30].

When considering a 20% additional margin for validation purposes, the total sample size required was 131 subjects, 26 at each of the five water depths analyzed. To reinforce the number of data points at both the extreme levels of immersion, we randomly assigned a minimum of 30 subjects to these levels and 25 to the three levels in the mid-range [31]. The 137 subjects who completed the protocol were randomly labeled as either a regression group (n = 113), which was used in a regression equation to predict GFR values; or a validation group (n = 24), which was used to assess the model performance independently.

The protocol for this study was approved by the Ethical Committee for Research on Humans of the University of the State of Santa Catarina. Informed consent was obtained from all participants, and their rights were protected.

Dependent variables were the peak values of the vertical (Fy) and antero-posterior (Fx) components of GRF as well as resultant force (F_R_) in Newtons (N), which is the result when summing other GRF components. We opted to not analyze the medio-lateral component separately due to great variability and the absence of a clear pattern [32].

The independent variables used in the prediction of GRF were velocity (m/s), water depth (m), body mass (kg), height, (m) and waist, thigh and leg circumferences (cm). The inclusion of these parameters was based on the current evidence provided in the literature [11,12,14,20,21,33–35] and the ease of measuring these parameters in a non-laboratorial setting. Circumference measures were of interest as a proxy for volume and frontal area [13].

Data for the GRF was collected (1000 Hz) with one force plate built with water proof strain gauges (dimensions 400×400×100 mm, maximum load/sensitivity of 4000/2 N, 300 Hz of natural frequency and an error of less than 1%) [14,21,33]. The force plate was placed at the bottom of a swimming pool (28° ± 1° C). A walkway of 8.4 meters was used, with the force plate fixed to a wooden frame support at 5 meters from the beginning and 3 meters from the end of the walkway. The walkway and platform were covered by a non-slipping material.

The variations in water depth (75, 90, 105, 120, e 135 cm) were achieved by moving the walkway up and down on the inclined pool floor (2.2 ± 0.2 degrees). The structure of the walkway and support compensated for the inclination of the pool floor such that the surface was perfectly horizontal. Walking velocity was measured and controlled using a system composed of lasers and photoresistors. Velocity and GRF were measured using a data acquisition system that included a signal conditioner, an A/D convertor and signal analysis and editing software (ADS2000-IP and AqDados 7.02, Lynx Tecnologia Eletrônica, São Paulo, Brazil).

Anthropometric data were acquired as follows: (a) body mass of the subjects using an electronic scale (model MEA-08128; Plenna Especialidades LTDA, São Paulo, Brazil; scale of 0.1 kg), (b) height of the subjects using a stadiometer (Sanny American Medical do Brasil LTDA, São Bernardo do Campo, Brazil; scale of 0.01 m), and (c) subjects’ circumferences using a measuring tape (Sanny American Medical do Brasil LTDA, São Bernardo do Campo, Brazil; TR4010, scale of 0.001). Thigh and leg circumferences were measured in the right limb. All measurements were performed by a well-trained and experienced researcher, who followed the guidelines of the International Society for Advancement in Kinanthropometry ISAK [36].

Following anthropometric measurements, subjects opened an envelope containing the water depth randomly selected and were invited to enter the pool. Subjects were given 10 to 15 minutes to familiarize themselves with the walkway and the water depth. While familiarization, the velocity of movement and the pattern presented were assessed by an experienced researcher and when both pattern and velocity remained stable and the subject felt comfortable with the task, trials were initiated.

At the given water depth, gait was analyzed at a self-selected velocity, which had been determined as the average value of three passages after familiarization. The following instruction was given: “Please walk at your comfortable velocity, without using your arms to propel yourself”. The arms were naturally aligned beside the trunk. Trials were considered valid if: i) the velocity was within the specific range (± 10% of self-selected velocity), ii) a double support phase was observed, iii) only one foot touched the force plate and iv) no propulsion movement was performed with the upper limbs.

Four hundred and eleven force curves were collected for each GRF component. With familiarization, subjects reached one valid trial after one or two attempts. After data collection, curves were exported through the software AqDAnalysis 7.0.14 (Lynx Tecnologia Eletrônica LTDA) to the software Scilab 4.1.2 (INRIA) where a processing routine was used. Data were low pass filtered at 20 Hz using a recursive third order Butterworth filter. Peaks (Fy, Fx and F_R_) were identified as the maximum value of the force curve during the contact time of each step. The raw data for prediction (S1 File) and validation (S2 File) can be found in Supporting Information.

Firstly, an exploratory analysis was performed to analyze data distribution and explore the relationship between variables using Pearson correlation and residual assessment. No data transformation was required since all independent variables were linearly scattered across dependent variables. The data of six subjects were excluded because they were beyond 3 standard deviations of the mean values obtained for the regression group. In addition, three data points (one for each dependent variable) were excluded from the analysis for the same reason.

The regression equations for F_R_, Fy and Fx were computed using a stepwise multiple regression model. Entering and exiting criteria of p≤0,05 and p>0,10, respectively, were used. Auto-correlation of the residual was tested to exclude possible temporal correlations.

Co-linearity between height and water depth was present and therefore a third variable based on the ratio between water depth and height was created–the immersion ratio. This variable is dimensionless and results from the quotient between water depth (m) and height (m).

The data from the validation group (n = 24) were used to validate the models. The data for the prediction model (named predicted) were compared to data collected with the force plate (named experimental). Predicted values of Fy, Fx and F_R_ were compared to the experimentally measured data using Student t-test, intra-class correlation (ICC estimates based on a mixed-effects model, absolute-agreement and single measures) and a graphical analysis of residual and agreement. Standard error of measurement (SEM) was computed as $SEM=SD\sqrt{1-ICC}$. All statistical procedures were performed using Statistical Package for the Social Sciences, SPSS 20.0.

## Results

In Table 1 and Table 2, mean and standard deviations of independent variables are shown.

**Table 1:** Mean and standard deviations of mass, height, circumferences and immersion ratio for the validation and regression groups.

Variables	Regression Group	Validation group
	(n = 113)	(n = 24)
**Mass (kg)**	68.7±12.5	67.5±14.2
**Height (m)**	1.71±0.08	1.71±0.09
**Waist circumference (cm)**	81.2±8.8	82.7±10.5
**Thigh circumference (cm)**	51.3±4.1	50.2±5.5
**Leg circumference (cm)**	32.9±3.3	32.2±7.2
**Immersion ratio**	0.61±0.12	0.60±0.11

kg = kilograms; m = meters; cm = centimeter

**Table 2:** Mean and standard deviations of gait velocity (m/s) at the different water depth levels for the validation and regression groups.

Water depth (m)	Regression Group		Validation group	
	N	velocity	n	velocity
0.75	27	0.73±0.13	5	0.72±0.17
0.90	14	0.66±0.12	5	0.62±0.10
1.05	25	0.62±0.11	5	0.62±0.06
1.20	19	0.52±0.11	5	0.49±0.11
1.35	28	0.47±0.11	4	0.49±0.14

m: meter; m/s: meter per second

**Fig 1:**
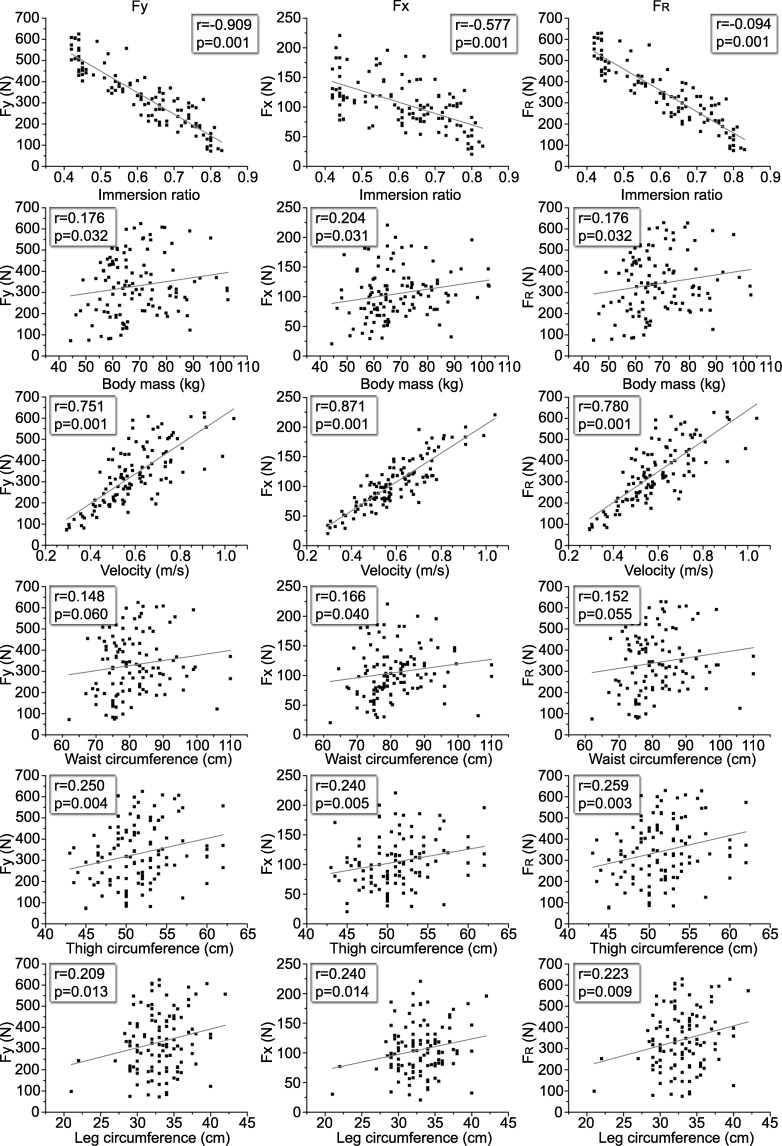
Pearson correlation scatter plot of dependent variables. Left: Fy = vertical component. Middle: Fx = antero-posterior component. Right: F_R_ = resultant force. Ground reaction forces by independent variables: immersion ratio, body mass, velocity, waist circumference, thigh circumference, leg circumference. (r = Pearson correlation coefficient, p = p-values).

The dispersion and correlation coefficients of the relationships between independent variables and Fx, Fy and F_R_ are shown in Fig 1.

In Table 3, the results of the multiple regression analysis are shown for Fy, Fx and F_R_.

**Table 3:** Estimated of the multiple regression analysis for the prediction of vertical (Fy), antero-posterior (Fx) and resultant (F_R_) of ground reaction force.

GRF	Variable	Coefficient	95% CI	Beta	p	R^2^
Fy	(Constant)	566.682	492.122 to 641.242			0.95
	Immersion ratio	-922.059	-993.245 to -850.873	-0.826	0.001	
	Body mass	3.321	2.795 to 3.847	0.295	0.001	
	Velocity	176.369	117.774 to 234.964	0.190	0.001	
Fx	(Constant)	-164.160	-208.002 to -120.318			0.81
	Velocity	243.626	221.009 to 266.243	0.872	0.001	
	Thigh Circumference	2.443	1.634 to 3,252	0.244	0.001	
F_R_	(Constant)	512.292	443.142 to 581.442			0.95
	Immersion ratio	-870.592	-936.612 to -804.571	-0.779	0.001	
	Body mass	3.331	2.843 to 3.819	0.295	0.001	
	Velocity	232.391	178.047 to 286.734	0.250	0.001	

R^2^ = adjusted determination coefficient; CI = confidence interval; immersion ratio = ratio between water depth and subject’s height.

The models result in the following equations:

Model for the prediction of the vertical component of GRF

$$F_{Y}=566.682-922.059\times (IMMERSION\;RATIO)+3.321\times \left(MASS\right)+176.369\times \left(VELOCITY\right)$$ (1)

Model for the prediction of the antero-posterior component of GRF

$$F_{X}=-164.160-243.626\times \left(VELOCITY\right)+2.443\times \left(THIGH\right)$$ (2)

Model for the prediction of the resultant GRF

$$F_{R}=512.292-870.592\times (IMMERSION\;RATIO)+3.331\times \left(MASS\right)+232.391\times \left(VELOCITY\right)$$ (3)

Where: **Immersion ratio**, water depth to height ratio; ***Mass***, body mass in kg; ***Velocity***, velocity in m/s; and ***Thigh***, subject's thigh circumference in cm.

**Fig 2:**
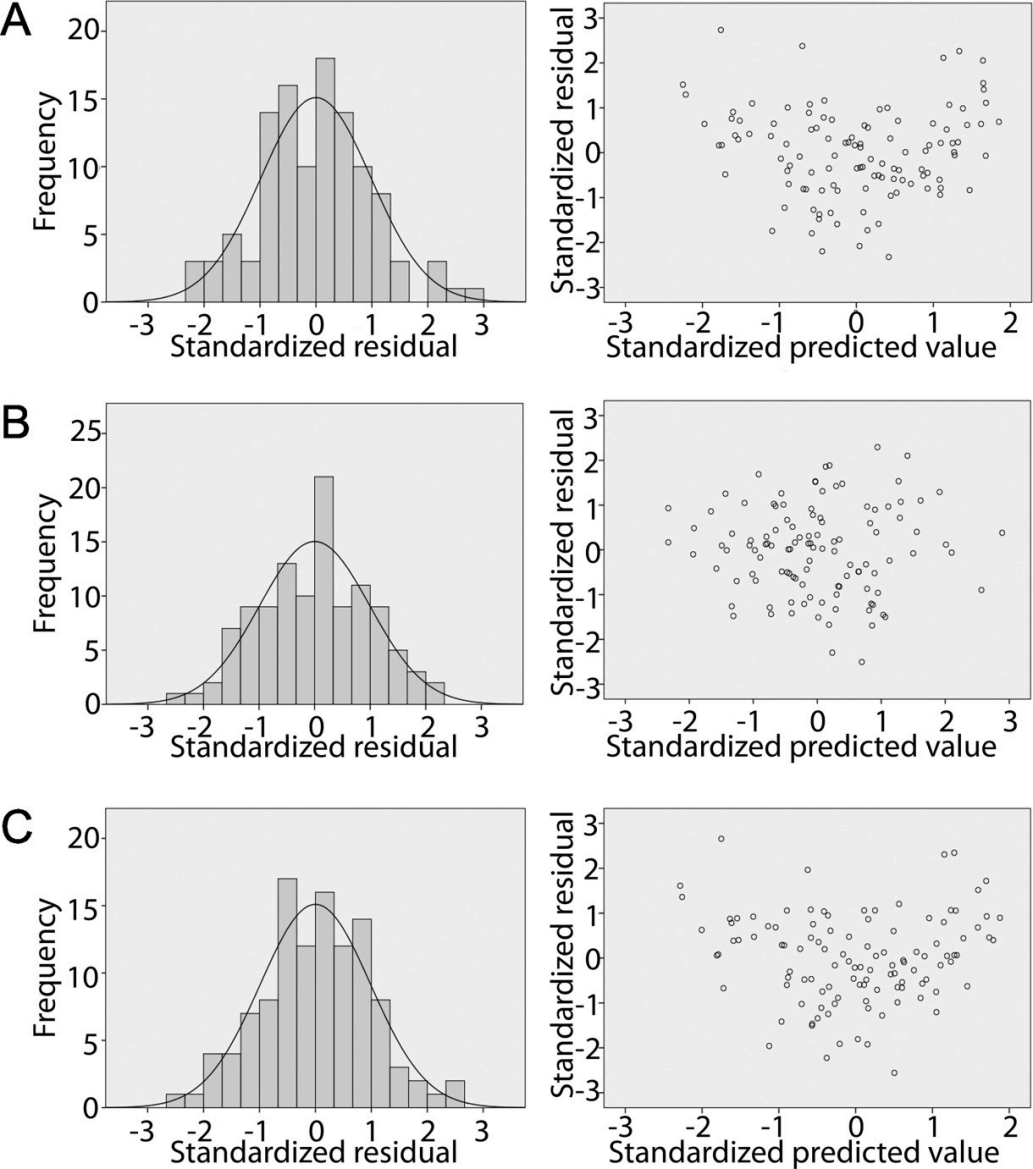
Residual analysis of the regression models. (A) Fy = vertical component. (B) Fx = antero-posterior component. (C) F_R_ = resultant force. Left: Residuals histogram. Right: Dispersion of residuals across predicted values.

Residual frequency and distribution across predicted values are in Fig 2. A normal distribution of residual was confirmed using Kolmogorov-Sminrnov (p>0.05).

In Table 4, the results of the comparison between the predicted and experimentally measured values of Fy, Fx and F_R_ for the validation group (n = 24) are shown. A good agreement was observed, with no significant differences between values (p = 0.84 to Fy, p = 0.19 to Fx and p = 0.81 to F_R_) and an ICC that varied between 0.89 and 0.90 depending on the condition.

**Table 4:** Comparison between the predicted and experimentally measured values of vertical (Fy), antero-posterior (Fx) and resultant (F_R_) of ground reaction force for the validation group (n = 24).

Variable	Standard deviation		P	ICC (95%)	SEM
	Experimental	Predicted			
**Fy (N)**	339.8 (133.0)	337.3 (122.3)	0.84	0.888 (0.759–0.950)	42.3
**Fx (N)**	107.2 (38.6)	102.7 (37.8)	0.19	0.903 (0.792–0.957)	11.8
**F**_**R**_ **(N)**	350.8 (132.8)	347.9 (123.0)	0.81	0.895 (0.773–0.953)	41.0

N: Newton.

**Fig 3:**
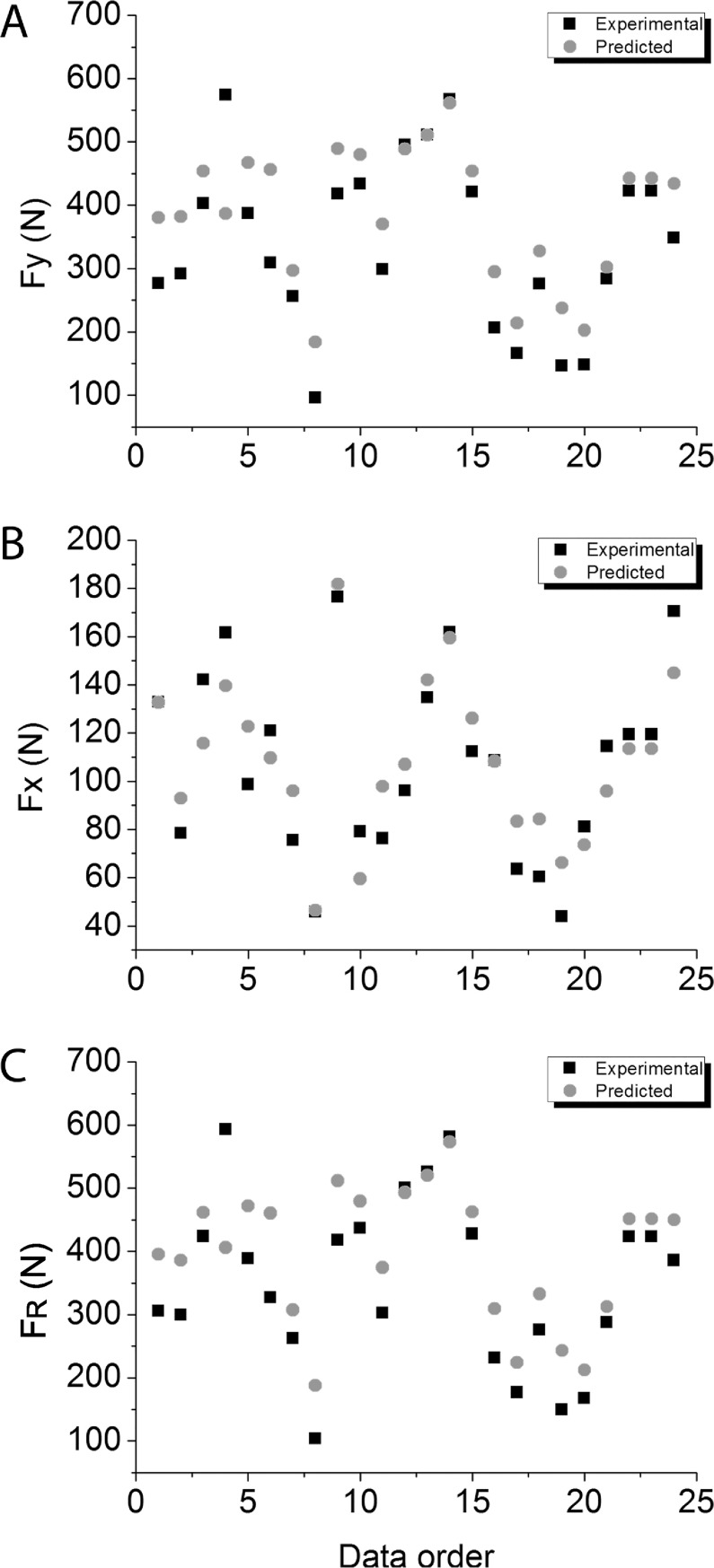
Comparison between mean data of predicted (gray circle) and experimentally measured (black square) values. (A) Vertical component. (B) antero-posterior component. (C) Resultant force.

In Fig 3, graphical comparisons between individual predicted and experimentally measured data points from the validation group are shown across all conditions for Fx, Fy and F_R_.

## Discussion

We aimed to develop statistical models to predict peak values of the vertical and antero-posterior components (Fx and Fy) as well as the resultant force (F_R_) while walking in water. The variables used in this study included velocity, immersion ratio and anthropometric measurements as significant predictors of GRFs and showed good predictive performance. Predicted values showed good agreement with those experimentally measured with the force plate and residuals were normally distributed, independent of predicted values and presented significant homoscedasticity (homogeneity of variance).

A regression model, besides being convenient in the estimation of variables that cannot be easily measured, is a useful tool for understanding a phenomenon [37]. Previous studies have developed models for the prediction of GRF while walking on dry land using velocity as predictor [38–40]. To allow independent variables to be easily measured and increase the applicability of the model, it is important that the model is as simple as possible [41].

The main predictor for Fy and F_R_ was the immersion ratio and for Fx, velocity. Eighty one percent of the variability of Fx could be predicted based on movement velocity and thigh circumference, while 95% of the variability of Fy and F_R_ was predicted based on immersion ratio, mass and walking velocity.

Two of the parameters entered in the models can be strategically manipulated by the professional prescribing the water exercise: the immersion ratio and movement velocity. The relationship between water depth and GRF has already been investigated in the literature in a variety of exercises [14,16,21,22,42]. Our results corroborate with the literature [11]. Roesler et al. found a significant increase in Fy when subjects were moved from a water depth at the manubrium sterni to a water depth at the xiphoid process. According to the regression model in our study, for a given mass and velocity, one can expect a variation of nearly 100N when immersion ratio is changed by 10% [11].

Commonly, different water depth levels are used to control loading in water but it is apparent that the height of the subject is as important as the water level used. Health professionals commonly control water depth when prescribing walking in water, often not considering that this will be dependent on the height of the subject. Obviously, a depth of 1 m will result in a different immersion ratio for an individual of 1.60 m than 1.85 m. The preferred load should be translated to an appropriate immersion ratio and movement speed in clinical practice.

In our study, velocity was shown to influence both GRF components, with a greater effect on Fx than on Fy. To our knowledge, this is the first study to report a significant relationship between velocity and Fy while walking in water at a self-selected velocity. This highlights the importance of understanding the variations in velocity that result from a given standardized instruction such as “walk at a comfortable” velocity. Roesler et al did not find a significant increase in Fy between a “slow” (metronome controlling cadence) and a “fast” (as fast as possible) gait but reported Fx values considerably higher for the fast gait [11]. The effect of velocity on Fx in water seems to be greater than that observed on land [43]. On land, an increase in velocity from 1 m/s to 2.5 m/s leads to an approximate increase of 100 N in the antero-posterior force peaks [38,40]. Although subjects walked at a much slower velocity in water, an increase of only 0.45 m/s in velocity resulted in a similar increase. Velocity can be easily manipulated by the physiotherapist and provides an alternative to control GRF when it is not possible to alter water depth.

This greater effect of velocity in water needs to be carefully considered during rehabilitation., The water provides a more viscous medium than the air and this enhances the effect of velocity on drag forces, which is exponential [13]. While buoyancy force reduces the apparent body weight during walking in water, the increased drag leads to an increase of the forces necessary to propel the body forward against the water’s resistance. This effect is expected to be more pronounced at the hip level, due to its role in propelling the lower limb forward during gait [44] leading to a smaller advantage of water walking–in terms of torque reduction–in individuals with hip injuries [34].

The presence of body mass and thigh circumference in the model highlights the effect of individual anthropometric characteristics on the magnitude of force required to move in water. These characteristics, although not modifiable, should be considered when walking in water is individually prescribed. Based on the model estimates, for a given velocity and water depth, a variation of approximately 33 N in Fy may be expected between individuals with a 10 kg difference in body mass. Thigh circumference was the determinant anthropometric parameter for Fx. Based on this model, a variation of approximately 10 N in Fx may be expected in individuals with a difference in thigh circumference of 5 cm. This relationship is possibly due to the greater magnitude of drag resulting from the greater frontal body projected area while forward displacement [13].

This study’s results are a step towards better control of GRF while walking in water for people undergoing rehabilitation, but future studies should focus on specific populations to further understand how injury may affect the parameters included in the model and its coefficient of determination. The models developed here are valid for the population studied and for the exercise conditions evaluated [45,46]. Therefore, the results are directly applicable to a healthy, adult population walking in water at the immersion ratio and velocities tested here (Tables 1 and 2).

Although this may be a limitation of this study, the changes in ground reaction force that result from immersing the body in water or from changing water exercise parameters (velocity and immersion) are generally more controllable and specific than those observed due to lower limb injuries [6,7]. If we consider subjects with similar anthropometric data and similar kinematics (parameters that were represented in the model) GRFs are expected to be similar.

Although GRFs are closely related to the mechanical loading during an exercise, they do not offer insight into coactivation levels or the way forces are distributed internally or how body tissues biologically respond. These parameters should be kept in mind when prescribing walking in water for rehabilitation.

Finally, the floor of the walkway was covered with a non-slipping material. If these characteristics are not considered, greater errors than the ones reported here are likely to occur. Future studies should focus on models for the prediction of GRF in populations with specific injuries or disabilities.

## Conclusion

Statistical models introduced here may be used as valid and precise tools to predict the magnitude of GRFs while walking in water. To control GRF while waking in water, the physiotherapist should control the velocity and water depth carefully, as small changes in these factors have an important effect on GRF components. The depth of immersion and velocity should be considered in the context of individual anthropometric characteristics.

## Supporting information

S1 File Data for the regression prediction. (CSV)

S2 File Data for the validation. (CSV)
